# Functional Interplay Between Posterior Parietal Cortex and Hippocampus During Detection of Memory Targets and Non-targets

**DOI:** 10.3389/fnins.2020.563768

**Published:** 2020-11-03

**Authors:** Elisa Ciaramelli, Hana Burianová, Antonino Vallesi, Roberto Cabeza, Morris Moscovitch

**Affiliations:** ^1^Department of Psychology, University of Bologna, Bologna, Italy; ^2^Center for Studies and Research in Cognitive Neuroscience, Cesena, Italy; ^3^Department of Psychology, Swansea University, Swansea, United Kingdom; ^4^Centre for Advanced Imaging, University of Queensland, Brisbane, QLD, Australia; ^5^Padova Neuroscience Center and Department of Neuroscience, University of Padua, Padua, Italy; ^6^Fondazione Ospedale San Camillo IRCCS, Venezia, Italy; ^7^Department of Psychology, Duke University, Durham, NC, United States; ^8^Department of Psychology, University of Toronto, Toronto, ON, Canada; ^9^Rotman Research Institute, Toronto, ON, Canada

**Keywords:** episodic memory, recognition memory decision, posterior parietal cortex, hippocampus, functional magnetic brain imaging (fMRI)

## Abstract

Posterior parietal cortex is frequently activated during episodic memory retrieval but its role during retrieval and its interactions with the hippocampus are not yet clear. In this fMRI study, we investigated the neural bases of recognition memory when study repetitions and retrieval goals were manipulated. During encoding participants studied words either once or three times, and during retrieval they were rewarded more to detect either studied words or new words. We found that (1) dorsal parietal cortex (DPC) was more engaged during detection of items studied once compared to three times, whereas regions in the ventral parietal cortex (VPC) responded more to items studied multiple times; (2) DPC, within a network of brain regions functionally connected to the anterior hippocampus, responded more to items consistent with retrieval goals (associated with high reward); (3) VPC, within a network of brain regions functionally connected to the posterior hippocampus, responded more to items not aligned with retrieval goals (i.e., unexpected). These findings support the hypothesis that DPC and VPC regions contribute differentially to top-down vs. bottom-up attention to memory. Moreover, they reveal a dissociation in the functional profile of the anterior and posterior hippocampi.

## Introduction

The ability to recollect specific past events, or episodic memory, depends on the interplay between the bottom–up emergence of stored memory traces and the top–down control of this process according to retrieval goals (e.g., Henson et al., 1999; Ranganath et al., 2000; Dudukovic and Wagner, 2007; Han et al., 2010; Quamme et al., 2010; Scimeca and Badre, 2012; Preston and Eichenbaum, 2013; Moscovitch et al., 2016). Functional neuroimaging (fMRI) and neuropsychological research have linked the bottom-up emergence of memories to the hippocampus, which has been characterized as a “stupid” module whose operations, once initiated, run obligatorily (Moscovitch, 2008). In contrast, top–down control retrieval processes have been attributed to the prefrontal cortex, deemed necessary to manage encoding and retrieval operations according to retrieval goals, while interacting with the hippocampus and associated medial temporal lobe (MTL) regions (Moscovitch and Winocur, 1992; Dobbins et al., 2002; Simons and Spiers, 2003).

More recent research has identified the posterior parietal cortex as another core element of the episodic retrieval network. In fMRI studies, posterior parietal cortex is one of the regions most frequently activated during episodic retrieval, and, critically, it almost always shows greater activity for successfully recognized old items (*hits*) than successfully rejected new items (*correct rejections—CRs*), or ‘retrieval success effect’ (Wagner et al., 2005; Cabeza et al., 2008; Ciaramelli et al., 2008). Moreover, patients with lesions to the posterior parietal cortex, though not amnesic, do show subtle anterograde and retrograde memory impairments (Berryhill et al., 2007; Davidson et al., 2008; Ciaramelli et al., 2010a,b; Simons et al., 2010; Ben-Zvi et al., 2015; Ciaramelli et al., 2017; Yazar et al., 2017). The posterior parietal cortex has long been associated with attention – not memory – and, therefore, there have been many attempts to explain the involvement of posterior parietal cortex in episodic memory retrieval (see, for reviews, Cabeza et al., 2008, 2012; Rugg and Vilberg, 2013; Gilmore et al., 2015; Humphreys and Lambon Ralph, 2017; Sestieri et al., 2017).

The ‘attention to memory’ hypothesis (Cabeza et al., 2008; Ciaramelli et al., 2008) sprang from the widely agreed observation that the two major divisions of posterior parietal cortex, dorsal parietal cortex (DPC; superior parietal lobule and intraparietal sulcus, roughly corresponding to BA7) and ventral parietal cortex (VPC; angular gyrus and supramarginal gyrus, roughly corresponding to BAs 39 and 40) have different functional profiles. DPC shows greater activity for low than high confidence memory judgments, when the engagement of memory search and top-down monitoring for diagnostic memory content is presumably maximal, whereas VPC is prominently active during the recognition of items of more obvious memory status, such as those accompanied by high confidence or the subjective feeling of recollection (Ciaramelli et al., 2008). The ventral/dorsal distinction observed in the memory domain echoes the distinction between the roles of DPC and VPC in attention: DPC supports top–down attention, which enables selection of stimuli based on internal goals, whereas VPC mediates the bottom-up capture of attention following detection of relevant stimuli (Marois et al., 2000; Corbetta and Shulman, 2002; Corbetta et al., 2008). Consistently, in the “Posner” paradigm, DPC is maximally engaged during the cue period, when participants search for a target, whereas VPC is engaged during target detection, and responds more strongly to invalidly compared to validly cued targets (Corbetta et al., 2000). According to the attention to memory model, DPC activity maintains retrieval goals, which modulate memory-related activity in the MTLs, whereas VPC activity mediates the change in the locus of attention following detection of relevant memories retrieved by the MTLs. A number of studies have provided empirical support to this model (reviewed in Cabeza et al., 2008, 2012). For example, in a cued recognition experiment, DPC was active when participants anticipated a target based on a memory cue, whereas VPC mediated fast detection of memory targets in the absence of cues (Ciaramelli et al., 2010a; see also Cabeza et al., 2011). Moreover, the left angular gyrus of VPC was more active during detection of invalidly vs. validly cued memory contents (Ciaramelli et al., 2010a; O’Connor et al., 2010; Jaeger et al., 2013).

Other studies have challenged the attention to memory model on a number of points. Hutchinson et al. (2009) pointed out that posterior parietal cortex subregions associated with top–down and bottom–up attention are adjacent but non-overlapping with those associated with episodic retrieval (Sestieri et al., 2017). Additionally, Hutchinson et al. (2014) detected multiple response profiles in posterior parietal cortex, of which only some appeared reflective of attention to memory. Therefore, although the dual attention system model was useful to frame the coarse segregation of DPC and VPC memory effects, this framework may not completely capture all the different functional properties of posterior parietal cortex subregions (Nelson et al., 2010; Sestieri et al., 2017). Although this debate is beyond the scope of the current article, we acknowledge that the number of studies specifically designed to test the attention to memory model has been limited, and these studies have employed paradigms that resembled attentional paradigms in some respect (e.g., use of cues, violation of expectations; Ciaramelli et al., 2010a; Cabeza et al., 2011).

To address this issue, the first goal of the current study was to test the attention to memory model using a standard recognition memory paradigm. We did so by manipulating two factors deemed to differentially affect bottom–up and top–down attention to memory: study repetitions and retrieval goals. During encoding, participants studied words either once (1x items) or three times (3x items), and during retrieval they were rewarded more either for detecting studied words (incentivize-old runs) or for detecting new words (incentivize-new runs). Regarding study repetitions, the attention to memory model assumes that VPC mediates bottom-up attention driven by salient memories, and hence it predicts greater VPC activity while detecting 3x than 1x items, as study repetition typically results in higher hit rates, shorter recognition times (e.g., Jacoby et al., 1998; Hembacher and Ghetti, 2014), and increased recognition confidence (Hembacher and Ghetti, 2014). In contrast, the model assumes that DPC mediates top-down attention required by demanding search and monitoring processes, and therefore it predicts greater DPC activity for 1x than 3x items. Regarding retrieval goals, the model assumes that DPC mediates top–down attention driven by retrieval goals, hence the strategic orienting of attention toward different classes of items (old, new) depending on payoffs. Thus, the model predicts greater DPC activity for detection of memory *targets* (i.e., events consistent with retrieval goals, because rewarded more: old items in incentivize-old runs and new items in incentivize-new runs) than *non-targets* (i.e., new items in incentivize-old runs and old items in incentivize-new runs). Conversely, the model assumes that VPC mediates the bottom-up capture of attention by salient events inconsistent with retrieval goals, and therefore predicts greater VPC activity for detection of non-targets than of memory targets, in line with previous evidence of VPC involvement in invalidly cued and involuntary memory retrieval (reviewed in Cabeza et al., 2008).

Although our main predictions pertain to posterior parietal cortex, highly complex cognitive processes, such as episodic memory retrieval, are expectedly mediated by the interaction among functionally related regions. Therefore, we adopted a multivariate method, Partial Least Squares (PLS) (McIntosh et al., 1997), to reveal the coordinated activity of distributed networks, supposedly including DPC and VPC, associated with top–down and bottom up attention to memory, respectively. In a previous work using the PLS method (Burianová et al., 2012), for example, we showed that DPC was functionally connected with a dorsal network of brain regions during cued (top–down) recognition memory trials (e.g., dorsolateral prefrontal cortex, precuneus), whereas VPC was functionally connected with a ventral network of brain regions during uncued (bottom–up) memory trials (e.g., ventrolateral prefrontal cortex, insula; Burianová et al., 2012). One important question pertains to the interaction between posterior parietal cortex (DPC and VPC) and the hippocampus. The hippocampus is thought to act as an index to neocortical structures representing the perceptual, conceptual, and emotional details of complex events (Teyler and Rudy, 2007; Stella et al., 2012). Recent research, however, indicates that differences exist in the type of information represented by the anterior and posterior hippocampi, based on their connectivity (Moscovitch et al., 2016). The posterior hippocampus is preferentially connected to perceptual regions in the posterior neocortex, supporting fine-grained, perceptually based memory representations, whereas the anterior hippocampus is preferentially connected to anterior regions, such as the ventromedial prefrontal cortex (vmPFC) and the amygdala, supporting memory representations that are more abstract (schematic) and subject to the influence of emotional/motivational processes (see Poppenk et al., 2013, for a review). Several studies have found evidence of connectivity between the posterior hippocampus and VPC (Uddin et al., 2010; Daselaar et al., 2013; Robin et al., 2015; Robin and Moscovitch, 2017), and we found evidence of anterior hippocampus-DPC connectivity in a study examining top–down attention to memory (Burianová et al., 2012). Based on this preliminary evidence, we predicted that the anterior hippocampus, which is connected with regions involved in motivation and reward processing, would be functionally coupled with DPC, responding more to either old or new items depending on which was rewarded more. In contrast, the posterior hippocampus, which is involved in the recollection of detailed memories (McCormick et al., 2015; Moscovitch et al., 2016), should be functionally coupled with VPC, and signal salient memories regardless of payoffs.

## Materials and Methods

### Participants

Fifteen young adults participated in the study (10 females), but a male subject was excluded due to reported discomfort in the scanner and consequently poor memory performance (*d*’ = −0.01). The final sample, therefore, comprised 14 young adults (age range 22–33, mean age 26 years), and the low sample size is one caveat of our study. For one participant, data from 2 out of the 8 recognition runs were lost due to a technical problem, and therefore data for this participant are relative to the remaining 6 runs. All participants were healthy, right-handed, English speakers, and with no psychiatric or neurological history. Participant received $60 to participate in the study, and an additional bonus of $30 depending on performance (see below).

### Stimuli and Procedure

Five hundred and twelve words (mean frequency = 25.49, *SD* = 34.9; mean concreteness = 4.92, *SD* = 1.79), between 4 and 13 letters long, were selected from the Kucera and Francis (1967) pool. Half of the words referred to concrete entities (e.g., volcano; mean concreteness = 6.56) and the other half referred to abstract entities (e.g., democracy; mean concreteness = 3.28). The words were subdivided into 4 lists of 64 concrete words (matched for frequency and concreteness; *p* > 0.69 in both analyses) and 4 lists of 64 abstract words (matched for frequency and concreteness; *p* > 0.98 in both analyses), which were randomly attributed to the different experimental conditions, with the study status (studied, unstudied) counterbalanced across participants.

The experimental paradigm was composed of a study phase and a test phase. During the study phase, outside the scanner, participants made concrete/abstract judgments on 256 words (128 abstract + 128 concrete), of which half were presented once (1x items) and the other half were presented three times (3x items). Each word was presented for 2800 ms followed by a fixation cross, which was presented for 200 ms. A scanned recognition memory test followed immediately afterward, which consisted of 8 runs. In each run, participants were presented with 64 words: 16 words studied once (8 abstract + 8 concrete), 16 words studied 3 times (8 abstract + 8 concrete), and 32 new words (16 abstract + 16 concrete), and had to recognize them as studied or new by pressing one of two keys (counterbalanced), located on an MRI-compatible response pad. The beginning of each recognition trial was signaled by a fixation cross that stayed in the center of the screen for 500 ms, and the target word was then presented for 3000 ms. Each recognition run also comprised 4 null events, in which a meaningless stimulus (i.e., xxxxxxx) appeared on the screen in the place of the word. Subjects were instructed to look at it and press one of the two response keys. An inter-trial-interval (ITI) (without fixation cross) that varied randomly between 2000 and 6000 ms was interspersed across test trials to “jitter” the onset times of trials and allow for event-related fMRI analyses.

In order to modify retrieval goals, and hence “memory targetness”, we used a payoff manipulation (Healy and Kubovy, 1978; Macmillan and Creelman, 1991). In half of the runs, subjects were informed that they would be rewarded 5 points for each correct “old” response and 1 point for each correct “new” response (i.e., incentivize-old runs). Conversely, in the other half, subjects were informed that they would be rewarded 5 points for each correct “new” response and 1 point for each correct “old” response (i.e., incentivize-new runs). Our assumption is that the manipulation would orient participants’ (top–down) attention toward different classes of items (old vs. new) depending on payoffs, with old words being the target for memory search in incentivize-old runs, and new words in incentivize-new runs. After each test run, the subject’s score for that run was displayed. Subjects were told that the participant with the highest final score would be rewarded an extra $30 after the experiment was completed, and $30 were accordingly awarded to the highest scoring subject. The order of incentivize-old and incentivize-new runs and the assignment of test words to the different runs were randomized for each participant.

### fMRI Data Acquisition and Pre-processing

Images were acquired at Baycrest Hospital on a 3 Tesla Siemens Magnetom Trio whole-body scanner with a matrix 12-channel head coil. Anatomical images were acquired using a MP-RAGE sequence (TR: 2 s, TE: 2.63 s, 160 oblique axial slices, with a 1 mm^3^ voxel size, FOV = 25.6 cm, acquisition matrix: 256 × 256). Brain activation was assessed using the blood oxygenation level dependent (BOLD) effect with optimal contrast (Ogawa et al., 1993). Functional images were obtained using a whole head T2^∗^-weighted echo-planar image (EPI) sequence (repetition time, TR: 2 s, echo time, TE: 30 ms, flip angle: 70°, 28 oblique axial slices with interleaved acquisition, 3.1 × 3.1 × 5 mm voxel resolution, field of view, FOV: 20 cm, acquisition matrix: 64 × 64). Physiological data (heart and respiration rate) were acquired during the scanning session.

The fMRI data were preprocessed using the Analysis of Functional NeuroImages software (AFNI; Cox, 1996). The initial five time points from each image volume were removed from analyses to allow for the brain magnetization to stabilize. EPI time-series data were corrected for cardiac and respiratory parameters (program 3dretroicor). Time-series data were spatially co-registered (program 3dvolreg) to correct for small head motion, using a 3-D Fourier transform interpolation. Each run was then normalized based on the mean intensity of the signal. Individual analysis was performed by generating the hemodynamic response function model for each condition, based on the convolution of the time points beginning with the stimulus presentation, using a block function (Cox, 1996). For each subject, 6 trial types of interest were modeled: (1) incentivize-old 3x hits, (2) incentivize-old 1x hits, (3) incentivize-old CRs, (4) incentivize-new 3x hits, (5) incentivize-new 1x hits, and (6) incentivize-new CRs. They were modeled by fitting a general linear model to the measured fMRI time series at each voxel (program 3dDeconvolve). The number of trials was > 22 in each of the 6 conditions of interest, for all subjects. Null events, false alarms, and misses were also modeled but were not used in the analyses. Prior to group analyses, the activation maps for each participant and each condition were spatially normalized to an average volume of 152 normal skull stripped brains. Datasets were then re-sampled with a 2 × 2 × 2 voxel dimension (program @auto_tlrc) and spatially smoothed with a 8 mm full-width half-maximum Gaussian kernel (program 3dmerge).

### fMRI Data Analysis

#### Whole-Brain Analysis

As memory processing is the result of integrated and coordinated activity of groups of brain regions (*i.e*., distributed brain networks) rather than the independent activity of any single brain region, fMRI data were analyzed with the Partial Least Squares multivariate analytical technique (PLS; McIntosh and Gonzalez-Lima, 1994; McIntosh et al., 1997; McIntosh and Lobaugh, 2004; for a detailed tutorial and review of PLS, see Krishnan et al., 2011), which is designed to identify groups of brain regions distributed over the entire brain whose activity changes as a function of task demands or is correlated with behavioral performance. PLS uses singular value decomposition (SVD) of a single matrix that contains all participants’ data to find a set of latent variables (LVs), which are mutually orthogonal dimensions that reduce the complexity of the data set. In the current study, we used whole-brain PLS to examine changes in activity in the six experimental conditions of interest. The output of PLS analysis is a set of LVs reflecting cohesive patterns of brain activity related to the experimental design, and accounting for maximum covariance between regional activity changes and task conditions. Thus, akin to Principal Component Analysis (PCA; e.g., Friston et al., 1993), PLS enables us to differentiate the degree of contribution of different brain regions associated with task or performance. Each LV consists of a singular image of voxel saliences (i.e., a spatiotemporal pattern of brain activity that reflects task-related changes or brain-behavior correlations seen across conditions), a singular profile of task saliences (i.e., a set of weights that indicate how brain activity in the singular image is expressed in each of the experimental conditions), and a singular value (i.e., the amount of covariance accounted for by the LV). The first LV always accounts for the largest amount of covariance (i.e., has the largest singular value), with subsequent LVs accounting for progressively smaller amounts. For each condition in each LV, we calculated summary measures of how strongly each participant expresses the particular pattern of activity seen on the LV. These measures, called brain scores, are the products of the weighted salience of each voxel and BOLD signals summed across the entire brain for each participant in each condition on a given LV (McIntosh and Lobaugh, 2004). Salience indicates the degree to which a voxel is related to the LV and can be positive or negative, depending on the relation of the voxel to the pattern of task-dependent differences identified by the LV. The significance and reliability of each LV was determined by permutation and bootstrap resampling tests (see below).

#### Functional Connectivity Analysis

In addition to whole-brain PLS analysis, we examined task-related functional connectivity (i.e., the degree of non-zero correlation between brain regions), using the ‘seed’ PLS analysis (McIntosh et al., 1997; Schreurs et al., 1997). Seed PLS is a multivariate statistical method widely used to investigate the relation between activity in a selected brain region (seed voxel) and activity in the rest of the brain, across task conditions (McIntosh et al., 1997; Schreurs et al., 1997; McIntosh, 1999; Della-Maggiore et al., 2000). Based on previous evidence on the differential role of the anterior and posterior hippocampus in episodic memory retrieval (Poppenk et al., 2013), and on the findings from the whole-brain PLS analysis, functional seed values were extracted from a region of interest with a neighborhood size of one voxel (i.e., including the seed voxel plus one voxel adjacent to the peak voxel in each direction; see also Marstaller and Burianová, 2015; Ziaei et al., 2017; Dzafic et al., 2019) centered in the left anterior hippocampus (MNI coordinates: *x* = −30, *y* = −10, *z* = −20) and the left posterior hippocampus (MNI coordinates: *x* = −26, *y* = −26, *z* = −22), to examine, respectively, task-related functional connectivity during detection of memory targets (i.e., including hits in the incentive-old condition and CRs in the incentive-new condition in the analysis) and detection of items that were not the target of memory (i.e., including hits in the incentive-new condition and CRs in the incentive-old condition in the analysis). The analytical procedure for the seed PLS functional connectivity analysis was the following: first, the BOLD values from the hippocampal seed regions were extracted for each event of interest (detection of memory targets and non-targets) across 8 time points from the onset of the trial. The activity for each seed region was averaged across the peak and adjacent timepoints, and then this average measure of seed activity was correlated with activity in all other brain voxels, across participants, within each condition of interest. These correlations were then combined into a matrix and decomposed with singular value decomposition (SVD), resulting in a set of LVs and voxel saliences.

The significance level for each LV is tested via two steps: permutations and bootstrap estimation, which is the standard analytical approach in PLS (e.g., McIntosh et al., 1997; Burianova and Grady, 2007; Vallesi et al., 2009; St-Laurent et al., 2011; Burianová et al., 2012, 2013; Ziaei et al., 2017; Corbett et al., 2020). The significance for each LV as a whole is determined using a permutation test (Edgington, 1980). The permutation test samples the distribution by resampling the observed data, testing the hypothesis of whether the whole-brain activity during a task/condition significantly differs from noise. At each permutation, the data matrix rows are randomly reordered and a new set of LVs is calculated each time. The singular value of each new LV is compared to the singular value of the original LV. A probability is assigned to the initial value based on the number of times a statistic from the permuted data exceeds this original value (McIntosh et al., 1997). For the current experiment, 500 permutations were used. If the probability was less than 0.05 then the LV was considered significant. This first step is then followed by a bootstrap test providing a direct assessment of the reliability of the significant patterns identified by the permutation test, and allows estimating voxel saliences, which are weights indicating how strongly a given voxel contributes to a significant LV. To determine the reliability of the saliences for the voxels characterizing each pattern identified by the LVs, all data were submitted to a bootstrap estimation of the standard errors, by randomly re-sampling subjects with replacement 100 times. PLS is recalculated for each bootstrap sample to identify those saliences whose value remains stable regardless of the sample chosen (Sampson et al., 1989). The ratio of the salience to the bootstrap standard error (bootstrap ratio, BSR) is approximately equivalent to a *z* score given a normal bootstrap distribution (Efron and Tibshirani, 1985). Peak voxels with a BSR > 3 (approximately equivalent to a *z*-score corresponding to *p* < 0.001) were considered as reliable. Since in PLS multivariate methods the whole-brain spatiotemporal patterns are derived in a single analytical step (via SVD, McIntosh and Lobaugh, 2004), there is no need for correction for multiple comparisons.

## Results

### Behavior

**FIGURE 1:**
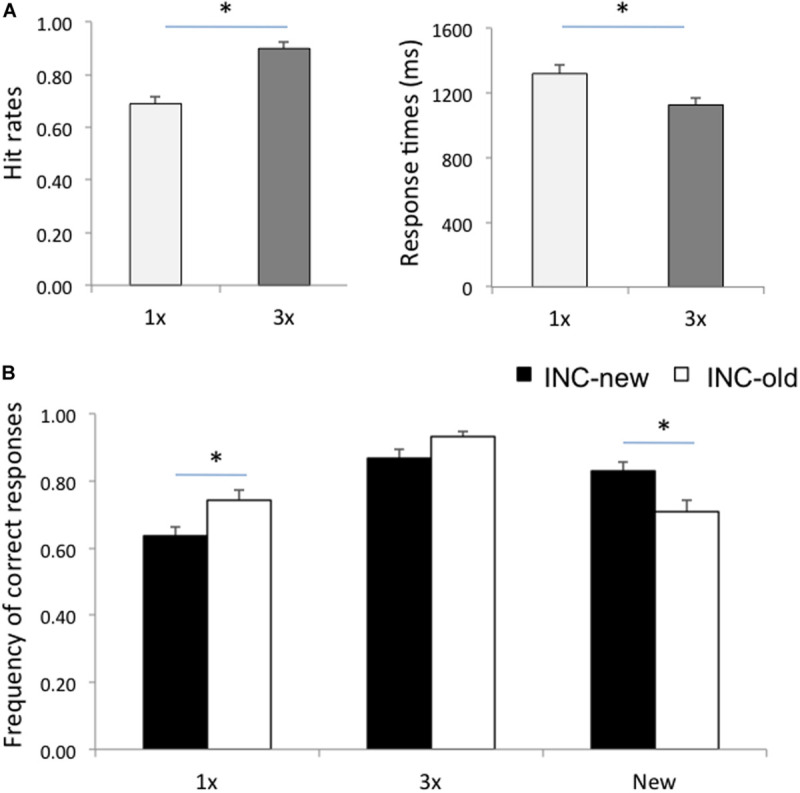
**(A)** Hit rates and response times for items studied once (1x) and for items studied three times (3x). **(B)** Frequency of correct responses for 1x, 3x, and new items in the Incentive-old (INC-old) and in the Incentive-new (INC-new) conditions. Error bars represent standard errors of the mean. Asterisks denote a significant difference (*p* < 0.05).

Behavioral results are summarized in Table 1 and Figure 1. An analysis of variance (ANOVA) on the frequency of correct responses with Item (1x, 3x, new) and Run (incentivize-old, incentivize-new) as within-subject factors yielded an effect of Item, *F*(2,26) = 38.10, *p* < 0.001, η_p_^2^ = 0.75, qualified by a significant Item X Run interaction, *F*(2,26) = 19.28, *p* < 0.001, η_p_^2^ = 0.60. *Post hoc* comparisons, performed with the Scheffè test, indicated that, as expected, hit rates for 3x items were higher than hit rates for 1x items (Figure 1A) in both the incentivize-old and incentivize-new condition (*p* < 0.001 in both cases). This result confirms the effectiveness of our study repetition manipulation. Importantly, hit rates for 1x items were higher in the incentivize-old compared to the incentivize-new condition (*p* = 0.02), whereas CR rates were higher in the incentivize-new than in the incentivize-old condition (*p* = 0.01). This result suggests that participants changed their retrieval orientation depending on whether hits or CRs were rewarded more, confirming the effectiveness of our targetness manipulation (Figure 1B). By contrast, hit rates for 3x items were comparable between incentivize-old and incentivize-new runs (*p* = 0.39). Arguably, the fact that participants had a very high recognition performance with 3x items rendered their ‘old’ status more obvious, and their recognition less sensitive to the payoff manipulation compared to 1x items (Figure 1B).

**TABLE 1:** Behavioral data.

	**Hit Rates**		**CR Rates**	**Sensitivity (d’)**	**Criterion (C)**	**RTs (correct responses)**		
	**1x**	**3x**	**New**			**1x**	**3x**	**New**
Incentivize-old condition	0.74 (0.03)	0.93 (0.02)	0.71 (0.03)	1.62 (0.14)	−0.22 (0.07)	1271 (54)	1089 (44)	1424 (67)
Incentivize-new condition	0.63 (0.02)	0.86 (0.02)	0.83 (0.03)	1.72 (0.17)	0.16 (0.05)	1383 (62)	1165 (48)	1371 (56)

*1x, items studied once; 3x, items studied 3 times; CR, correct rejection; RTs, response times. The values in parenthesis are standard errors of the mean.*

A similar ANOVA on response times (RTs) for correct responses showed an effect of Item, *F*(2,26) = 41.60, *p* < 0.001, η_p_^2^ = 0.76, qualified by a significant Item X Run interaction, *F*(2,26) = 7.07, *p* = 0.003, η_p_^2^ = 0.35. *Post hoc* Scheffè comparisons showed that individuals were faster at recognizing 3x than 1x items (Figure 1A), in both the incentivize-old and incentivize-new conditions (*p* < 0.001 in both cases), again confirming the efficacy of our study repetition manipulation (Figure 1A). RTs for correctly recognizing 1x items (p = 0.07), 3x items (p = 0.39), and new items (*p* = 0.74) did not change significantly between the incentivize-old and incentivize-new conditions, although participants tended to be faster at recognizing 1x items in the incentivize-old condition (when they were the target of memory search; see Table 1).

We also report the estimates of response bias and sensitivity (collapsing hit rates for 1x items and 3x items; see Table 1). Response bias was estimated with a criterion location measure, defined as c = 0.5[z(H) + z(F)] (Macmillan and Creelman, 1991). Negative c values indicate a liberal response bias, whereas positive values indicate a conservative response bias, and expectedly participants exhibited lower c values in the incentivize-old than in the incentivize-new condition, *t*(13) = 4.86, *p* < 0.001. In contrast, sensitivity, estimated as *d* = z(H) – z(F) (Macmillan and Creelman, 1991), did not differ significantly between conditions, *t*(13) = 1.24, *p* = 0.23. A two-one-sided test for equivalence (TOST; Lakens, 2017), however, indicated that the observed effect size (Cohen’s dz = −0.26) was not significantly within the equivalence bounds of dz = −0.50 and dz = 0.50, and therefore it was not statistically consistent with a lack of a medium effect-size result, *t*(13) = 0.89; *p* = 0.196, though it could exclude a large effect-size result, *t*(13) = 2.01; *p* = 0.033.

### fMRI

#### Whole-Brain Analysis

We report patterns of brain activity related to study repetitions (including 3x hits vs. 1x hits) and retrieval goals (including memory targets, i.e., hits in the incentive-old condition and CRs in the incentive-new condition, vs. non-targets, i.e., hits in the incentive-new condition and CRs in the incentive-old condition). In the targetness analysis, we included only 1x items, because the mnemonic status of these items is less obvious and more influenced by criterion manipulations than that of 3x items, consistent with the results obtained on hit rates (see Table 1 and Figure 1B; see also Stretch and Wixted, 1998; Aminoff et al., 2012, for similar findings). Including all hits led to a similar pattern of results.

##### Study repetitions

**FIGURE 2:**
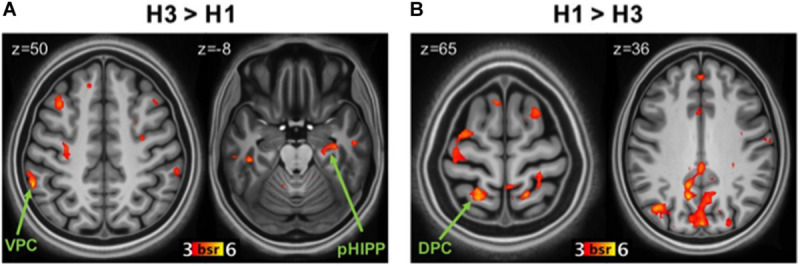
Brain regions related to study repetitions. **(A)** Regions more active during correct recognition of 3x items (H3) than 1x (H1). Panel **(B)**: regions more active during H1 than H3. DPC, dorsal parietal cortex; VPC, ventral parietal cortex; pHIPP, posterior hippocampus; BSR, salience/standard error ratio from the bootstrap analysis.

The statistically significant LV (*p* = 0.038) delineated a whole pattern of brain regions that responded differentially to 3x and 1x hits (Figures 2A,B). In line with previous research (e.g., Kim and Cabeza, 2007; Cabeza et al., 2008; Ciaramelli et al., 2008; Rugg and King, 2018), detection of items studied three times vs. once was associated with activity in VPC (supramarginal gyrus; Figure 2A) bilaterally (*p* < 0.001; see Table 2 for the complete list of activations). Consistent with our hypotheses, the 3x study repetitions pattern also included the right posterior hippocampus. Activity was also detected in the parahippocampal gyri and a network of brain regions including the anterior and lateral prefrontal cortex. In contrast, in line with previous evidence (e.g., Kim and Cabeza, 2007; Cabeza et al., 2008; Ciaramelli et al., 2008), detecting items studied only once was associated with activity in the left DPC (superior parietal lobule), although activity in the angular gyrus bilaterally was also detected (Figure 2B). This pattern of brain activity also included a more anterior region of the left hippocampus, as well as the medial and lateral prefrontal cortex and the anterior cingulate cortex (see Table 2).

**TABLE 2:** Networks related to study repetitions.

**Area**	**BA**	***X***	***Y***	***Z***	**BSR**
**3x HITs > 1x HITs**					
Ventral parietal cortex (supramarginal gyrus)	40	−56	−44	50	6.01
	40	58	−36	46	3.01
Posterior hippocampus		20	−36	8	3.28
Parahippocampal gyrus	36	−44	−32	−16	4.92
	35	32	−22	−26	5.47
Frontopolar cortex	10	34	46	12	5.26
Dorsolateral prefrontal cortex	46	−40	38	8	4.08
	9	48	−2	18	4.73
Lateral temporal cortex	21	−66	−38	−8	4.18
	21	58	−22	−12	3.77
	41	50	−36	4	4.75
Insula	13	−36	6	16	3.46
	13	50	6	−2	3.55
**1x HITs > 3x HITs**					
Dorsal parietal cortex	7	−12	−52	68	4.23
Ventral parietal cortex (angular gyrus)	39	−36	−70	30	4.40
	39	36	−64	36	5.73
Middle/posterior Hippocampus		−37	−24	−12	4.42
Parahippocampal gyrus	36	38	−28	−18	6.20
Medial prefrontal cortex	9	−6	52	14	4.74
	10	−2	56	0	3.48
	10	−28	50	6	3.41
Dorsolateral prefrontal cortex	9	40	22	32	3.22
Ventrolateral prefrontal cortex	47	−38	38	−6	4.27
Anterior cingulate cortex	24	−24	−6	36	3.28
	24	6	−16	38	4.37
Posterior cingulate cortex	31	−22	−64	18	4.27
	23	4	−34	22	5.85
Lateral temporal cortex	21	−56	−12	−20	4.92
	22	−46	−22	4	5.47
	38	42	2	−22	5.71

*Key: 1x HITs, hits to items studied once; 3x HITs, hits to items studied three times; BA, Brodmann area; BSR, salience/standard error ratio from the bootstrap analysis. We report Montreal Neurological Institute (MNI) coordinates.*

##### Targetness

**FIGURE 3:**
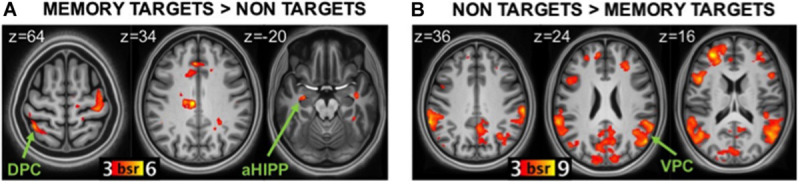
Brain regions related to memory targetness. **(A)** regions more active during detection of memory targets (hits in the Incentive-old condition and CRs in the Incentive-new condition) than non-targets (CRs in the Incentive-old and hits in the Incentive-new condition). **(B)** regions more active during detection of non-targets than memory targets. DPC, dorsal parietal cortex; VPC, ventral parietal cortex; aHIPP, anterior hippocampus; BSR, salience/standard error ratio from the bootstrap analysis.

The statistically significant LV (*p* = 0.046) delineated a whole pattern of brain regions that responded differentially to detection of memory targets and non-targets (Figures 3A,B). Consistent with our hypotheses, detection of memory targets was associated with activity in the left DPC (superior parietal lobule and precuneus; Figure 3A), along with a relatively dorsal region of the inferior parietal lobule bilaterally (*p* < 0.001; see Table 3 for the complete list of activations). The ‘targetness pattern’ also included the anterior hippocampus, and the ventrolateral prefrontal and anterior cingulate cortex. A different pattern of brain regions evinced higher activation for items that were not in line with retrieval goals. Consistent with our hypotheses, these included prominently VPC, with multiple peaks in the inferior parietal lobule and supramarginal gyrus, in addition to the precuneus (Figure 3B). The left posterior hippocampus was also activated, along with a more anterior region of the right hippocampus, and the medial and dorsolateral prefrontal cortex (see Table 3).

**TABLE 3:** Networks related to memory targetness.

**Area**	**BA**	***x***	***y***	***z***	**BSR**
**Memory targets > Non-targets**					
Dorsal parietal cortex	7	−36	−50	64	4.30
Precuneus	7	−12	−54	50	5.39
Ventral parietal cortex (inferior parietal lobule)	40	−52	−48	48	4.48
	40	36	−46	48	4.47
Anterior hippocampus		−30	−10	−20	3.95
Ventrolateral prefrontal cortex	47	−38	50	−10	4.44
Anterior cingulate cortex	32	−4	30	34	5.82
Lateral temporal cortex	21	−62	−42	−12	4.61
Temporo-occipital cortex	37	−38	−58	−12	6.26
Occipital cortex	19	−28	−70	6	5.09
	19	24	−82	6	6.60
Insula	13	−26	10	12	4.56
**Non-targets > memory targets**					
Ventral parietal cortex (supramarginal gyrus)	40	−62	−40	24	5.56
	40	56	−42	24	4.75
	40	−64	−42	36	6.05
	40	64	−40	36	5.61
Precuneus	7	6	−60	32	4.66
Posterior hippocampus		−26	−26	−22	5.49
Anterior/middle hippocampus		20	−20	−20	3.35
Medial prefrontal cortex	10	10	48	−6	4.70
Dorsolateral prefrontal cortex	46	−30	46	16	8.74
	46	44	46	10	4.42
Lateral temporal cortex	21	−48	−38	4	6.06
	21	48	−8	−10	5.56
Temporo-occipital cortex	37	−54	−64	6	4.86
	37	58	−66	12	9.64

*Key: Memory targets, hits in the Incentive-old condition and CRs in the Incentive-new condition; Non-targets, hits in the Incentive-new condition and CRs in the Incentive-old condition; BA, Brodmann area; BSR, salience/standard error ratio from the bootstrap analysis. We report Montreal Neurological Institute (MNI) coordinates.*

#### Functional Connectivity of the Hippocampus During Detection of Memory Targets and Non-targets

To test our hypothesis that the left anterior hippocampus would be functionally connected to DPC during detection of memory targets, and the left posterior hippocampus would be functionally connected to VPC during detection of non-targets, we investigated their task-related functional connectivity during detection of memory targets (hits in the liberal condition and CRs in the conservative condition) and non-targets (hits in the conservative condition and CRs in the liberal condition). The results confirmed that the left anterior hippocampus was functionally connected to the DPC during detection of memory targets but not during detection of non-targets (*p* < 0.001; see Table 4 for the complete list of activations). Regions functionally connected to the anterior hippocampus for target detection also included VPC (supramarginal gyrus), the anterior cingulate cortex, and the ventrolateral prefrontal cortex. These functional associations were significant for hits in the incentive-old condition, but not for CRs in the incentive-new condition. By comparison, the left posterior hippocampus was functionally connected to VPC (inferior parietal lobule and supramarginal gyrus) bilaterally during detection of items that were not the target for memory search (including both hits in the incentive-new condition and CRs in the incentive-old condition) but not during detection of memory targets (*p* < 0.001; see Table 4 for the complete list of activations). Regions functionally connected to the posterior hippocampus also included the dorsolateral prefrontal cortex and the right anterior hippocampus. The supramarginal gyrus of VPC exhibited a significant positive correlation with the left posterior hippocampus for CRs in the incentive-old condition, but a negative correlation for hits in the incentive-new condition, as did the dorsolateral prefrontal cortex, suggesting a selective engagement in signaling unexpected novelty (see also Jaeger et al., 2013). Because detection of non-targets also entailed activity in a right anterior hippocampal region, for completeness we ran the same functional connectivity analysis using this region as the seed (Table 3). The anterior hippocampus seed was functionally connected to the left posterior hippocampus (*p* < 0.001), but less connected to the anti-targetness network itself: for hits in the incentive-new condition, but not for CRs in the incentive-old condition, it was functionally associated with the right supramarginal gyrus, the inferior parietal lobule, and the left dorsolateral prefrontal cortex, but showed a negative correlation with the left supramarginal gyrus and the right dorsolateral prefrontal cortex.

**TABLE 4:** Hippocampal functional connectivity.

**Area**	**BA**	***x***	***y***	***z***	**BSR**
**Detection of memory targets**					
Anterior hippocampus (*seed*)		−30	−10	−20	3.95
Dorsal parietal cortex	7	−36	−50	64	4.30
Ventral parietal cortex	40	36	−46	48	4.47
Anterior cingulate cortex	32	−4	30	34	5.82
Ventrolateral prefrontal cortex	47	−38	50	−10	4.44
**Detection of non-targets**					
Posterior hippocampus (*seed*)		−26	−26	−22	5.49
Anterior/middle hippocampus		34	−20	−20	3.35
Ventral parietal cortex	40	−62	−40	24	5.56
	40	56	−42	24	4.75
	40	−64	−42	36	6.05
Dorsolateral prefrontal cortex	46	−30	46	16	8.74
	46	44	46	10	4.42

*BA, Brodmann area; BSR, salience/standard error ratio from the bootstrap analysis. We report Montreal Neurological Institute (MNI) coordinates.*

## Discussion

The first goal of the present study was to test the attention to memory model of posterior parietal contributions to episodic memory retrieval during a standard recognition memory task by manipulating study repetitions, and supposedly the saliency of recovered memories at retrieval, and memory goals, hence memory targetness. The second goal of the study was to investigate the functional connectivity of posterior parietal regions during bottom–up and top–down attention to memory, and, in particular, the interaction between posterior parietal regions and the posterior and anterior hippocampus. We had three main predictions. First, we predicted that VPC would be more active for detection of 3x than 1x items, whereas DPC would be more active for 1x than 3x items. Second, we predicted that DPC would be more active for detection of memory targets than non-targets, whereas VPC would be more active for non-targets than targets. Finally, we predicted that VPC connectivity would be stronger with the posterior hippocampus, whereas DPC connectivity would be stronger with the anterior hippocampus. The results were generally consistent with our predictions, but there were also some unpredicted findings.

### Effect of Study Repetitions

Consistent with attention to memory model (Cabeza et al., 2008; Ciaramelli et al., 2008), correct recognition of 3x items (vs. 1x) was associated with increased activity in the supramarginal gyrus of VPC, consistent with the hypothesis that VPC signals retrieval of salient memories that capture attention in a bottom–up fashion (Cabeza et al., 2012), and not in DPC. Items that have been studied multiple times are indeed generally recognized quickly and with high confidence (Jacoby et al., 1998; Hembacher and Ghetti, 2014), which is also associated with the engagement of VPC (Kim and Cabeza, 2007; Ciaramelli et al., 2008; Hayes et al., 2011; Rugg and King, 2018). Also consistent with the attention to memory model, hits for 1x (vs. 3x) items were associated with increased DPC activity: 1x items likely passed through more pre- and post-retrieval processing before being endorsed as old, requiring the sustained deployment of attentional resources (Cabeza et al., 2008; Sestieri et al., 2010; Hutchinson et al., 2014).

One unexpected result was the finding of greater activity for 1x than 3x hits in the angular gyrus within VPC. An influential hypothesis maintains that the angular gyrus acts as an episodic buffer to hold integrated representations retrieved from episodic memory in the service of memory decisions (Baddeley, 2003; Vilberg and Rugg, 2007; Guerin and Miller, 2011; Shimamura, 2011; see also Bonnici et al., 2016; Ramanan et al., 2018). Activity in VPC, indeed, has been found to increase with the amount of information recollected (Vilberg and Rugg, 2007, 2009). It is possible, therefore, that the less obvious mnemonic status of 1x than 3x items made them more behaviorally relevant, because more susceptible to the payoff manipulation, as borne out in the behavioral data (Figure 1). Therefore, activity in the angular gyrus may reflect the prolonged online maintenance of 1x memories needed to integrate memory signals with payoffs in order to drive adaptive decisions and earn points, as suggested by increased RTs (Table 1). This interpretation is compatible with the view that the angular gyrus supports an episodic buffer for retrieved information in the service of memory decisions, whereas the supramarginal gyrus mediates effects more directly related to bottom-up attention (Hutchinson et al., 2014; Sestieri et al., 2017).

### Effect of Targetness

Consistent with the attention to memory model (Cabeza et al., 2008; Ciaramelli et al., 2008), DPC (superior parietal lobe and precuneus) was sensitive to memory targetness, responding strongly to hits in the incentive-old condition and CRs in the incentive-new condition, consistent with a role in top-down attention to memory. The successful retrieval of memory targets was also marked by activity in a dorsal, anterior region of the inferior parietal lobe, along with nodes of the salience network (Seeley et al., 2007), such as the anterior cingulate cortex and the insula.

This finding makes contact with previous studies showing that activity in the posterior parietal cortex is related to response bias (Miller et al., 2001; O’Connor et al., 2010; Aminoff et al., 2015). A common finding of these studies is that DPC regions respond more to recognition hits when studied items are infrequent (vs. frequent), which typically results in a more conservative criterion (Herron et al., 2004; Vilberg and Rugg, 2009; Aminoff et al., 2015; King and Miller, 2017). Note that the task used in most previous studies required detecting *studied* words (memory targets; Aminoff et al., 2015), and when studied items are few, their behavioral relevance increases further. DPC may thus index the behavioral relevance (targetness) of retrieved items (Platt and Glimcher, 1999; Assad, 2003), be this determined by mnemonic expectations (Vilberg and Rugg, 2009; Aminoff et al., 2015; King and Miller, 2017; see also O’Connor et al., 2010) or payoffs (this study). In particular, we argue that DPC mediated the top-down orienting of attention toward different classes of items (old, new) depending on payoffs, consistent with reduced RTs for memory targets compared to non-targets (as is observed for valid trials in the Posner task). Our results are also consistent with previous evidence that posterior parietal cortex tracks retrieval goals, even though this is not consistently confined to the DPC (e.g., Dudukovic and Wagner, 2007; Quamme et al., 2010). For example, Favila et al. (2018) found that DPC -but not VPC- represented more goal-relevant than goal-irrelevant feature information at retrieval. Kuhl et al. (2013) found that DPC and the supramarginal gyrus (but not the angular gyrus) were more active during goal-relevant vs. incidental reactivation of event features. Han et al. (2010) found that a VPC region responded more to hits when incentives were paired with old compared to new recognition memory decisions. With respect to the striatum, however, our findings diverged from those of Han et al. (2010; for review see Scimeca and Badre, 2012, and references therein), in that we did not find differential striatal responses depending on targetness. This discrepancy may be related to the fact that our design manipulated the amount of reward associated with hits and CRs (1 vs. 5 points), but did not contain a condition with no reward, or with punishment (unlike Han et al., 2010).

A different neural network was engaged when participants detected items that were not the target of memory search, because not in line with retrieval goals. In this case, we observed bilateral activity in multiple sites of VPC, including the supramarginal gyrus and a ventral region of the inferior parietal lobule, and no activity in DPC. This finding is consistent with the hypothesis that VPC signals the bottom–up reorienting of attention to salient yet unattended memories, and aligns with previous evidence that VPC activity is associated with unintentional memory retrieval (LaMontagne and Habib, 2010; Guerin and Miller, 2011; Hall et al., 2014), with retrieval of items that were invalidly (vs. validly) cued (Ciaramelli et al., 2010a; O’Connor et al., 2010; Jaeger et al., 2013), with retrieval of items overcoming active suppression (Benoit and Anderson, 2012), and even with mind-wandering, the automatic drift of attention away from an external task toward inner thoughts (e.g., memories; Andrews-Hanna et al., 2010), which consistently engages VPC but not DPC (Fox et al., 2015).

### Parietal-Hippocampal Connectivity

The results show that the left anterior hippocampus was associated with the detection of memory targets, along with DPC, whereas the left posterior hippocampus was associated with the detection of targets not aligned with memory goals, along with VPC. The response profiles of the anterior and posterior hippocampi are consistent with the recently described functional organization along the hippocampal antero-posterior axis, according to which the anterior hippocampus supports coarse memory representations, subject to the influence of schematic knowledge and motivational factors, whereas the posterior hippocampus supports fine-grained representations related to recollection abilities (Poppenk and Moscovitch, 2011; Poppenk et al., 2013; Strange et al., 2014; McCormick et al., 2015; Robin et al., 2015; Bonnici and Maguire, 2018). In our study, indeed, the anterior hippocampus was influenced by retrieval goals rather than its objective memory status, whereas the posterior hippocampus supported memory decisions not influenced by (in fact, in conflict with) retrieval goals. Other studies have found that motivational salience modulates activity in the anterior hippocampus (Kumaran and Maguire, 2006; Zweynert et al., 2011). In other words, the ‘stupidity’ quality provocatively attributed to the hippocampus by Moscovitch (2008) to describe the obligatory nature of retrieval (Moscovitch and Winocur, 1992) appears to apply to its posterior sector only, as the anterior hippocampus can be made to care about mnemonic goals and reward.

Other regions functionally connected to the anterior hippocampus in signaling targetness were a dorsal region of the anterior cingulate cortex, which has been associated with cognitive control (Carter et al., 1998; Braver et al., 2001; di Pellegrino et al., 2007), and the ventrolateral prefrontal cortex, which has been linked with the selection of task-relevant memory contents (Badre and Wagner, 2007). These regions were likely necessary to monitor participants’ retrieval goals along with the objective memory status of items, in order to favor rewarding response strategies. The anterior hippocampus was also functionally connected with a region in the right inferior parietal lobe, possibly mediating detection of task-relevant memory contents. The functional connectivity of the left posterior hippocampus involved, in addition to VPC regions, a more anterior region of the right hippocampus, perhaps encoding the ‘contextual novelty’ of items violating mnemonic expectations (Daselaar et al., 2006; Kumaran and Maguire, 2006; Martin et al., 2011), and a dorsal prefrontal region widely implicated in post-retrieval evaluation of memory output with respect to task relevance and accuracy (Rugg et al., 1999; Rossi et al., 2001; Fleck et al., 2006).

In conclusion, our results show that DPC, within a network of brain regions functionally connected to the anterior hippocampus, is associated with top–down attention to memory retrieval, supporting retrieval of items consistent with memory goals and of items of uncertain memory status. By comparison, VPC, within a network of brain regions functionally connected to the posterior hippocampus, is more prominently associated with retrieval of salient memories, and of retrieval cues not aligned with the current goals and mental sets, which both capture attention bottom–up.

### Limitations

A caveat of this study is the small sample size. It would be important, therefore, to confirm our finding of a differential role of DPC and VPC in top–down and bottom–up attention to memory with larger samples, or to seek complementary, causative evidence for this dissociation, for example testing patients with focal lesions to DPC or VPC or interfering with these regions with transcranial magnetic stimulation (TMS).

In addition, although our results are generally consistent with a dorsal/ventral functional partition of posterior parietal cortex during episodic memory retrieval, we have also found evidence, as other have (Hutchinson et al., 2009, 2014; Sestieri et al., 2017), that VPC does not behave as a single functional unit. In the present study, for example, the supramarginal and the angular gyrus responded preferentially during detection of items studied multiple times and once, respectively. Thus, overarching single-function accounts of VPC, such as the attention to memory model (Cabeza et al., 2012), will need to be modified, or supplemented, to take such findings into account. On the other hand, we note that a similar functional heterogeneity characterizes the attentional properties of VPC. For example, TMS evidence shows that the angular (but not the supramarginal) gyrus is critical for reorienting attention after invalid cueing (Rushworth et al., 2001; Chambers et al., 2004; see also Ciaramelli and Moscovitch, 2020). Future studies will clarify whether posterior parietal cortex has multiple mnemonic properties or, rather, episodic memory retrieval engages multiple facets of attention.

## Data Availability Statement

The data that support the findings of this study are available from the corresponding author upon reasonable request.

## Ethics Statement

The studies involving human participants were reviewed and approved by Roman Research Institute. The patients/participants provided their written informed consent to participate in this study.

## Author Contributions

EC, MM, and RC conceived the study. EC, HB, and AV collected and analyzed the data. EC wrote the first draft of the manuscript. All the authors edited the manuscript and approved its final version.

## Conflict of Interest

The authors declare that the research was conducted in the absence of any commercial or financial relationships that could be construed as a potential conflict of interest.
